# Case Report: Preimplantation Genetic Testing for Meckel Syndrome Induced by Novel Compound Heterozygous Mutations of *MKS1*

**DOI:** 10.3389/fgene.2022.843931

**Published:** 2022-03-14

**Authors:** Tingting Lin, Yongyi Ma, Danni Zhou, Liwei Sun, Ke Chen, Yezhou Xiang, Keya Tong, Chaoli Jia, Kean Jiang, Dongyun Liu, Guoning Huang

**Affiliations:** ^1^ Chongqing Key Laboratory of Human Embryo Engineering, Chongqing, China; ^2^ Chongqing Clinical Research Center for Reproductive Medicine, Chongqing, China; ^3^ Reproductive and Genetic Institute, Chongqing Health Center for Women and Children, Chongqing, China; ^4^ The Southwest Hospital of Army Medical University, Chongqing, China

**Keywords:** *MKS1* gene, Meckel syndrome, PGT-M, intron mutation, exon skipping variant

## Abstract

Meckel syndrome (MKS), also known as the Meckel–Gruber syndrome, is a severe pleiotropic autosomal recessive developmental disorder caused by dysfunction of the primary cilia during early embryogenesis. The diagnostic criteria are based on clinical variability and genetic heterogeneity. Mutations in the *MKS1* gene constitute approximately 7% of all MKS cases. Herein, we present a non-consanguineous couple with three abnormal pregnancies as the fetuses showed MKS-related phenotypes of the central nervous system malformation and postaxial polydactyly. Whole-exome sequencing identified two novel heterozygous mutations of *MKS1*: c.350C>A and c.1408-14A>G. The nonsense mutation c.350C>A produced a premature stop codon and induced the truncation of the MKS1 protein (p.S117*). Reverse-transcription polymerase chain reaction (RT-PCR) showed that c.1408-14A>G skipped exon 16 and encoded the mutant MKS1 p.E471Lfs*92. Functional studies showed that these two mutations disrupted the B9–C2 domain of the MKS1 protein and attenuated the interactions with B9D2, the essential component of the ciliary transition zone. The couple finally got a healthy baby through preimplantation genetic testing for monogenic disorder (PGT-M) with haplotype linkage analysis. Thus, this study expanded the mutation spectrum of *MKS1* and elucidated the genetic heterogeneity of *MKS1* in clinical cases.

## Introduction

Cilia are microtubule-based organelles that extend from the surface of most eukaryotic cells. Defects in this organelle cause a series of disorders known as ciliopathies (Mitchison and Valente, 2017; Reiter and Leroux, 2017; Andreu-Cervera et al., 2021; Luo et al., 2021). Meckel syndrome (MKS, MIM 249000) is a rare and lethal autosomal recessive ciliopathy with highly variable phenotypes, extreme genetic heterogeneity, and complex allelism with other related ciliopathies, such as Joubert syndrome (JBTS, MIM 213300) (Salonen et al., 1984a; Parisi, 2019). MKS is mainly characterized by central nervous system malformation (most commonly occipital encephalocele), cystic kidney dysplasia, fibrotic changes of the liver, and postaxial polydactyly (Logan et al., 2011; Hartill et al., 2017). Globally, the incidence rate of MKS has been estimated at 1/140,000–1/13,250 in live births, and a high prevalence was observed in Finland and Belgium (Salonen et al., 1984b; Auber et al., 2007). The MKS-affected fetuses usually die *in utero* or shortly after birth.

Genetic studies have identified several genes related to MKS, such as *MKS1*, *TMEM216*, *TMEM67*, *CEP290*, *RPGRIP1L*, *CC2D2A*, *NPHP3*, *TCTN2*, *B9D1*, *B9D2*, *TMEM231*, *KIF14*, and *TMEM107*, and most of them encode proteins concentrated to the ciliary transition zone (TZ) (Bergmann et al., 2016; Dean et al., 2016; Wu et al., 2020). The TZ is characterized by Y-shaped structures spanning from the axoneme to the ciliary membrane that functions as a barrier between the cilia components and the cytoplasmic group to regulate the material transport and signal transmission of the cilia (Garcia-Gonzalo et al., 2011; Anvarian et al., 2019). Systematic genetic studies have grouped the known TZ proteins into three functional modules: MKS module, NPHP module, and CEP290 module (Goncalves and Pelletier, 2017). The B9 domain-containing proteins, including MKS1, B9D1, and B9D2, function as soluble MKS-module components and are associated with normal cilia biogenesis and ciliary diffusion (Bialas et al., 2009; Gogendeau et al., 2020; Okazaki et al., 2020). Previous studies have identified >80 pathogenic *MKS1* mutations that contribute to approximately 7% of all reported MKS cases (Hartill et al., 2017).

Preimplantation genetic testing (PGT) is an invasive prenatal diagnosis that involves the biopsy of a single or few cells from *in vitro* fertilized embryos and testing of the biopsied samples for genetic aberrations, followed by the selective transfer of unaffected embryos under specific conditions (De Rycke and Berckmoes, 2020). Clinically, PGT is available for monogenic disorder (PGT-M), wherein the disease-causing locus has been identified unequivocally. In the present study, we identified two novel *MKS1* mutations, c.350C>A and c.1408-14A>G, in a couple with three times of abnormal pregnancies. The genetic analysis and functional study showed the pathogenicity of these two mutation sites. Finally, assisted reproductive technology (ART) combined with PGT-M helped the couple get a healthy baby. These findings extended the spectrum of *MKS1* mutations in MKS and manifested the role of PGT in blocking single-gene diseases.

## Methods and Materials

### Subjects and Ethical Approval

The non-consanguineous couple first visited the Institute of Reproduction and Genetics, Chongqing Health Center for Women and Children (Chongqing, China), three times due to abnormal pregnancies and to consult for PGT. Pedigree data were obtained from the couple and their parents. Clinical assessments, including ultrasonic examination and assisted reproductive technology (ART), were performed in the related clinical departments. This study was approved by the Ethics Committee of the Chongqing Health Center for Women and Children. Informed consent was obtained from the couple.

### Whole-Exome Sequencing and Variants Analysis

The exomes were captured using the Agilent SureSelect Human All Exon V6 Kit (Agilent Technologies Inc., CA, United States) and sequenced on an Illumina NovaSeq 6000 platform (Illumina Inc., CA, United States). The clean reads derived from targeted sequencing were filtered and aligned to the human reference genome (GRCh37/hg19) using the Burrows–Wheeler Aligner (BWA) (Li and Durbin, 2009). Single-nucleotide variants (SNVs) and InDels were called using Genome Analysis Toolkit (GATK), annotated with Ensembl Variant Effect Predictor (McLaren et al., 2016), and filtered using multiple databases, including NCBI dbSNP, HapMap, 1,000 human genome dataset, and gnomAD. Finally, all the variants were annotated according to the guidelines of the American College of Medical Genetics and Genomics (ACMG) (Richards et al., 2015), and the variants from known causative genes of MKS were analyzed. The Human Gene Mutation Database (HGMD) and VarSome were used to screen the mutations reported previously. The variants were validated by Sanger sequencing.

### Validation of Mutations

**FIGURE 1:**
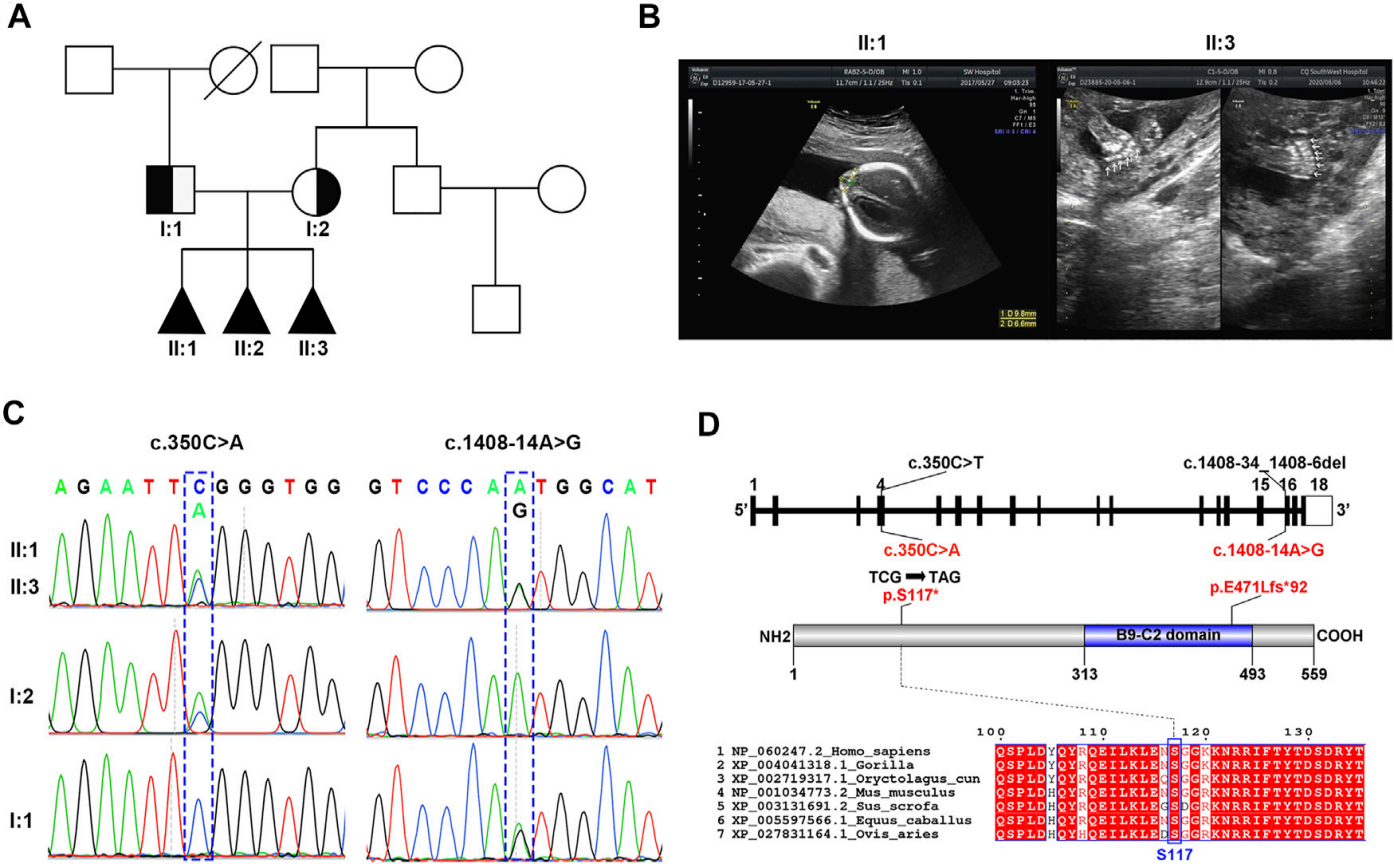
Identification of novel *MKS1* mutations in a Chinese family with MKS. **(A)** The pedigree of this family showed a history of abnormal pregnancy. **(B)** Ultrasonographic images of fetuses II:1 and II:3 showed the occipital encephalocele and postaxial polydactyly, respectively. **(C)** Sanger sequencing of *MKS1* showed the inheritance pattern of mutant sites between the couple and the three fetuses. **(D)** Schematic of *MKS1* gene and MKS1 protein. The mutation sites with related amino acid change were labeled. The B9 domain is labeled as the B9-C2 domain.

Sanger sequencing was used to validate *MKS1* mutations in the aborted fetuses (II:1 and II:3) and the couple (I:1 and I:2) (Figure 1A) The following primers were used: *MKS1*-exon4-forward: 5′-TTC​TTG​GTT​CCC​CTG​CCA​TTC-3′, *MKS1*-exon4-reverse: 5′-CTC​ACC​ACC​TGT​AGA​CTG​TGC-3’; *MKS1*-intron15-forward: 5′-CTG​TGT​CAT​TGC​TGG​GGA​GTC-3′, *MKS1*-intron15-reverse: 5′-CCA​GCC​ACA​TGG​TTA​CGG-3′. The products were purified on 2% agarose gels, sequenced with ABI 3500 (Thermo Fisher, MA, United States), and analyzed using Chromas 2.6.5 (Technelysium Pvt. Ltd., United States).

### Amino Acid Conservation and Protein Sequences

The amino acid sequence and name of mutant MKS1 proteins were analyzed using Name Checker (https://mutalyzer.nl/name-checker). The MKS1 amino acid sequences from different species were compared using the ClustalW software and analyzed using ESPript 3.0 (https://espript.ibcp.fr/ESPript). Wild-type and mutant MKS1 proteins were modeled using Illustrator for Biological Sequences (IBS, http://ibs.biocuckoo.org/online.php).

### Intron Mutation Analysis and Reverse-Transcription Polymerase Chain Reaction

The mutations at the 3′-terminus of intron 15 were found in the gnomAD database (http://gnomad-sg.org/). The effect of these mutations was analyzed with varSEAK (https://varseak.bio/) and SpliceAI (https://spliceailookup.broadinstitute.org/). Total RNA was extracted from blood samples using the QIAamp RNA blood mini kit (Qiagen, Germany), according to the manufacturer’s instructions. PrimeScript Reverse-Transcription Polymerase Chain Reaction (RT-PCR) kit (Takara, Japan) was used for RT-PCR. The forward primer (5′-GGC​TGA​GCT​GAG​GAG​GTT​TT-3′) used for cDNA amplification was located at exon 15, while the reverse primer (5′-CTT​CCA​GAC​GGT​CCA​ACA​CA-3′) was located at exon 17. Then, the products were purified on 2% agarose gels and analyzed on ABI 3500.

### qPCR

The primers used for real-time fluorescence quantitative PCR (qPCR) were as follows: forward 5′-CTC​CGA​GTC​CAC​CTG​CAA​AGA​ATC-3′ and reverse 5′-CTC​CTC​CTC​TTC​GTC​TTC​CTC​TGG-3′ for *MKS1* exons 2 and 3; forward 5′-GGA​TCC​TTC​AAG​GGG​GAA​CG-3′ and reverse 5′-CAT​GAA​GGC​CCT​GGA​CTG​CT-3′ for *MKS1* exon 16; forward 5′-ATG​CAG​AAT​CCA​CGC​CAG​TAC​AAG-3′ and reverse 5′-TCA​GTC​GCT​CCA​GGT​CTT​CAC​G-3′ for *RPS18* as the control. The expression of *MKS1* was evaluated using EvaGreen SuperMix (Bio-Rad, United States) on a CFX96 apparatus (Bio-Rad, United States) and analyzed using the 2^−ΔΔCt^ method by normalizing to that of *RPS18*.

### Plasmid Construction

The full-length coding sequence (CDS) of *MKS1* (NM_017777.4) was amplified by RT-PCR from wild-type, c.350C>A heterozygous, and c.1408-14A>G heterozygous subjects and subcloned into the pcDNA3.1-Myc B vectors (Invitrogen, USA). B9D2 was inserted into the pCMV-HA plasmid. All plasmid sequences were validated by Sanger sequencing.

### Cell Culture, Transfection, and Western Blot Analysis

HEK293T cells were provided by Stem Cell Bank, Chinese Academy of Sciences (Shanghai, China), and grown at 37°C in the presence of 5% CO_2_ in DMEM (HyClone, United States) supplemented with 10% fetal bovine serum (HyClone, United States). 293T cells were seeded in six-well plates (Corning, United States), and 2.5 µg wild-type or mutant MKS1 vector was transfected using Lipofectamine 3000 reagent (Thermo Fisher, United States). The cells were lysed with cell lysis buffer for western blot and immunoprecipitation (IP) (Beyotime, China) after transfection for different times (12, 24, 36, and 48 h), and 20 μg protein was analyzed by western blot. The anti-Myc antibody (AM926, 1:1,000) was purchased from Beyotime. Actin antibody (Beyotime, AA128, 1:1,000) served as an internal control.

### Co-IP

For co-IP, MKS1 (1.5 µg) and B9D2 (1.0 µg) expression vectors were co-transfected into HEK293T cells. After 36 h post-transfection, HEK293T cells were rinsed with ice-cold phosphate-buffered saline (PBS) and lysed with IP lysis buffer (Beyotime, China) supplemented with a protease inhibitor cocktail. After 20 min, cell lysates were cleared by centrifugation at 14,000 × *g*, 4°C for 5 min. The supernatant was used for the co-IP assay by shaking with BeyoMag™ anti-Myc magnetic beads (Beyotime, China) at 4°C for 4–6 h. After three washes, protein-Myc bead complex was eluted with IP buffer containing 150 μg/ml 3× Myc peptides (Beyotime, China) for 2 h. Then, the elution products were subjected to western blotting. The following antibodies (1:1,000, Beyotime, China) were used: anti-Myc, anti-HA, and goat anti-mouse HRP.

### Controlled Ovarian Stimulation

The COS was conducted using a gonadotropin-releasing hormone (GnRH) antagonist protocol based on the ovarian reserve of the I:2 subject. It was initiated on day 2 of the cycle with a dose of 250 IU recombinant follicle-stimulating hormone (rFSH, Puregon, Organon, Netherlands). GnRH antagonist (0.25 mg; Cetrotide, Merck Serono, Switzerland) was given on cycle day 8. Human chorionic gonadotropin (hCG, Merck Serono, Switzerland) was administered as a trigger on cycle day 10, and transvaginal oocyte retrieval was performed after 36 h. Consequently, 13 oocytes were obtained, and five blastocysts were biopsied after intracytoplasmic sperm injection (ICSI).

### PGT-M Procedure

Whole-genome amplification of each embryo biopsy sample was performed using the MALBAC WGA kit (Yikon Genomics, China), following the manufacturer’s instructions. A total of 60 single-nucleotide polymorphism (SNP) markers linked to the mutation alleles were selected for linkage analysis. The mutation site and SNPs were amplified using specific primer pairs; the amplification products were pooled with the MALBAC WGA products and sequenced. The chromosomal copy number and the mutation site and SNPs were analyzed, as published previously (Huang et al., 2015).

## Results

### Identification of Novel *MKS1* Mutations in MKS-Related Family

As shown by the family genetic map (Figure 1A), the non-consanguineous couple (I:1 and I:2) suffered from abnormal pregnancy three times, while the family presented no related medical history. Ultrasonographic images showed that the three aborted fetuses (II:1, II:2, and II:3) had clinical features of MKS such as occipital encephalocele, cerebellar vermis agenesis, and postaxial polydactyly (Figure 1B; Supplementary Table S1). Renal/hepatic involvement was not observed by ultrasonography in these fetuses. WES of the proband II:3 identified two novel *MKS1* (NM_017777.4) variants, c.350C>A and c.1408-14A>G (Figure 1C). Moreover, analysis of *MKS1* with Sanger sequencing in I:1, I:2, II:1, and II:3 showed that these two compound heterozygous mutations were inherited from their parents (Figure 1C).

The maternally inherited variant, c.350C>A, is a novel nonsense mutation causing the premature stop of MKS1 translation at the conserved Ser117, thereby encoding the mutant MKS1 p.S117*, while the paternally inherited variant, c.1408-14A>G, is located at intron 15 (Figure 1D). *MKS1* c.350C>A is not recorded in the human disease-related databases (gnomAD, ClinVar, and HGMD), and the truncated MKS1 protein would be a loss of function without the C-terminal B9-C2 domain. *MKS1* c.1408-14A>G is known as rs1194131222 with a rare frequency (0.000007228) in the gnomAD database, and no clinical case has yet been reported. Thus, according to the ACMG guidelines, the *MKS1* c.350C>A mutation is classified as “pathogenic” (PVS1: very strong pathogenicity, PS4: strong pathogenicity, PM2: moderate pathogenicity, and PP1: supporting pathogenicity), and the *MKS1* c.1408-14A>G mutation is classified as “uncertain significance” (PM3+PP1+PP4).

### c.1408-14A>G Induced the Skip of Exon 16 in *MKS1* mRNA Splicing

**FIGURE 2:**
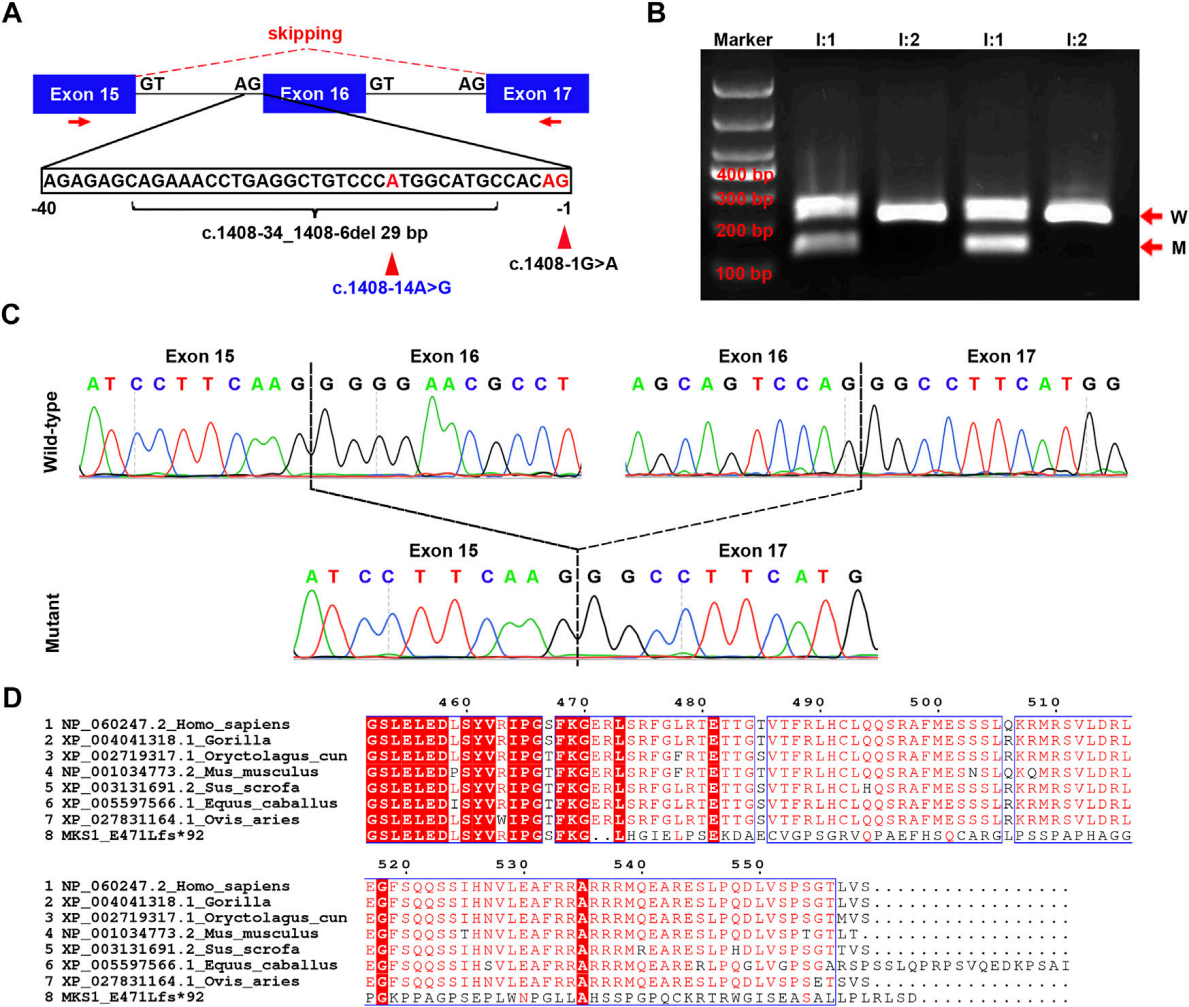
Confirmation for the effect of c.1408-14A>G on *MKS1* mRNA splicing. **(A)** Schematic of the three exons (exons 15, 16, and 17), the partial sequence of intron 15, and the location of three variants (c.1408-1G>A, c.1408-14A>G, and c.1408-34_1408-6del29bp). The red arrows indicate the localization of primers used in RT-PCR. **(B)** Image of agarose gel electrophoresis of the RT-PCR products from the couple (I:1 and I:2). The red arrows show the wild-type (W) and mutant (M) product. **(C)** Sanger sequence of the RT-PCR products. The upper panel shows the wild-type *MKS1* sequence with two dashed lines labeling the boundary of exon 15/exon 16 and exon 16/exon 17. In the mutant sequence, exon 15 was ligated directly with exon 17, manifesting the ship of exon 16 during mRNA splicing. **(D)** Alignment results of human wild-type and mutant MKS1 proteins with other species (gorilla, pig, horse, sheep, rabbit, and mouse). Only the C-terminal sequence is displayed.

As previously reported, *MKS1* c.1408-34_1408-6del29bp [AGAAACCTGAGGCTGTCCCAATGGCATGC], the Finnish major mutation, affected the *MKS1* mRNA splicing with the skip of exon 16 and induced frameshift of the MKS1 protein, resulting in MKS in a homozygous pattern (Kyttälä et al., 2006; Auber et al., 2007). Aberrant splicing was reported as a crucial mutational mechanism in *MKS1*-induced Meckel–Gruber syndrome (Frank et al., 2007). To date, 22 variants have been identified at the 3′-terminus of intron 15, while two variants (c.1408-1G>A and c.1408-34_1408-6del29bp) were predicted to induce the skip of exon 16, as assessed by varSEAK and SpliceAI (Figure 2A; Supplementary Table S2). The analysis showed an uncertain significance of c.1408-14A>G on *MKS1* mRNA splicing.

To further investigate the effect of c.1408-14A>G, RT-PCR was conducted with primers specific to exons 15 and 17. The results showed that there was one band >200 bp in I:2, and there were two bands in I:1 (one >200 bp and one <200 bp) (Figure 2B). Sanger sequencing of the two bands demonstrated a direct connection between exons 15 and 17 in the shorter one with the skip of exon 16 (Figure 2C), implying that the effect of c.1408-14A>G was like c.1408-34_1408-6del29bp on *MKS1* mRNA splicing. Using the *in silico* prediction software Name-Checker, the mutant CDS was predicted to encode the MKS1 p.E471Lfs*92 protein (Supplementary data S1). BLAST and alignment with MKS1 proteins across evolution manifested the partial dysfunction of the highly conserved B9-C2 domain (313–493 aa) in MKS1 p.E471Lfs*92 (Figure 2D). Herein, we updated the clinical significance of *MKS1* c.1408-14A>G as “pathogenic.”

### Mutations Disrupted the Function of MKS1 Protein

**FIGURE 3:**
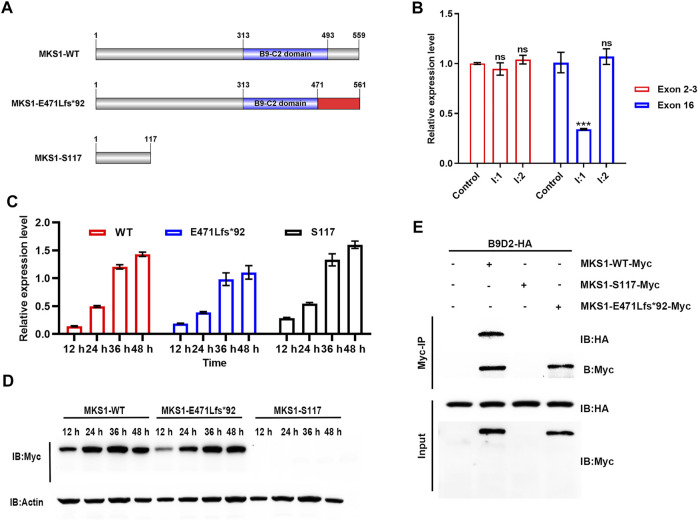
MKS1 mutants lose the interaction with B9D2 protein. **(A)** Schematic of the MKS1 proteins (wild-type, E471Lfs*92, S117). The red rectangle indicates the frameshifted sequence of MKS1 p.E471Lfs*92. **(B)** Relative *MKS1* mRNA levels of the individuals (I:1 and I:2) compared to the healthy control. **(C)** Relative *MKS1* mRNA levels of 293T cells transfected with Myc-tagged MKS1 CDS shown in **(A)** for 12, 24, 36, and 48 h. Data from three independent experiments were used for quantification. Error bars represent mean ± SD. Statistical significance was determined by unpaired Student’s *t*-test. ns: nonsense. ***p < 0.001. **(D)** Immunoblot analysis of 293T cells shown in **(C)**, implying the expression of wild-type and frameshifted MKS1 with the absence of MKS1 p.S117*. **(E)** Immunoprecipitation of Myc-tagged MKS1 variants with B9D2. The pull-down of B9D2 with the Myc bead demonstrated the function of the B9-C2 domain of MKS1 proteins.

Previous studies have shown that the nonsense codons in all internal exons could trigger a nonsense-mediated mRNA decay (NMD) process (Nagy and Maquat, 1998). The c.350C>A variant resulted in a premature stop codon in exon 4, which probably triggered the degradation of mutant *MKS1* mRNA through the NMD pathway, while the c.1408-14A>G variant induced the skip of exon 16 and generated a new stop codon in the 3′-untranslated region (3′-UTR) (Figure 3A). The results of qPCR with specific primers for exons 2–3 and 16 respectively showed that the expression level of *MKS1* gene in an I:2 heterozygous subject was equivalent to the control individual (Figure 3B), implying the absence of NMD process for the c.350C>A variant. Moreover, the *MKS1* level detected for exon 16 was downregulated in the c.1408-14A>G heterozygous individual (I:1), confirming the skip of exon 16 during the *MKS1* mRNA splicing process.

The C-terminal B9-C2 domain (313–493 aa) of MKS1 protein is conserved across evolution and essential for the predominant interaction between MKS1, B9D1, and B9D2, which is essential for cilial function (Dowdle et al., 2011; Romani et al., 2014; Okazaki et al., 2020). The truncated protein MKS1 p.S117* produced by the c.350C>A variant was predicted to lose its function completely, while the elongated protein MKS1 p.E471Lfs*92 maintained partial B9-C2 domain (313–470 aa) with the frameshift of the C-terminus (471–561 aa). We transfected 293T cells with Myc-tagged wild-type or mutant MKS1 expression plasmids to investigate the effect of these two variants on the B9 domain function. qPCR with specific primers for exons 2–3 confirmed the transcription of wild-type and mutant *MKS1* in the transfected 293T cells (Figure 3C). However, immunoblots with anti-Myc antibody demonstrated the expression of the wild-type and the frameshifted MKS1 proteins, while the MKS1 p.S117* was undetectable (Figure 3D). Co-IP assays with B9D2 protein showed that the wild-type MKS1 but not the MKS1 p.E471Lfs*92 interacted with B9D2, implying the dysfunction of the B9-C2 domain of MKS1 p.E471Lfs*92 (Figure 3E). Thus, c.350C>A and c.1408-14A>G variants disrupted the function of MKS1 and were pathogenic for fetal development.

### PGT for the *MKS1* Variants

**FIGURE 4:**
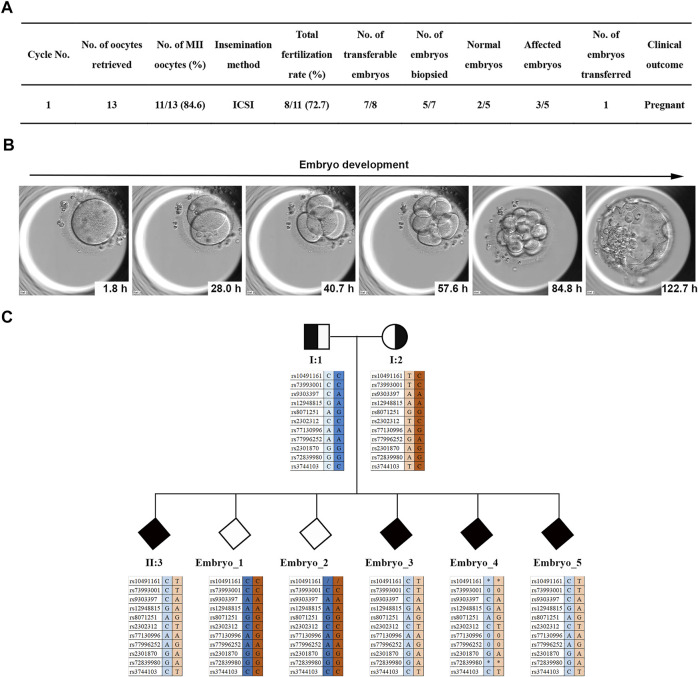
PGT for the two *MKS1* variants. **(A)** Statistical data of the ART and PGT-M cycles of the MKS-related couple. **(B)** The images of *in vitro* early embryonic development. **(C)** Results of haplotype linkage analysis. Based on the genotype of the SNP locus of the aborted fetus II:3, embryos carrying the *MKS1* gene mutations were deduced: unknown gender without *MKS1* mutations; unknown gender with *MKS1* mutations.

To avoid the occurrence of abnormal pregnancy, the non-consanguineous couple (I:1 and I:2) chose the *in vitro* assisted reproductive technology combined with PGT-M for the *MKS1* variants. Clinically, the I:2 individual was consecutively treated with an antagonist for ovulation induction, and 11 mature oocytes at the metaphase II (MII) stage were retrieved through the laparoscopic ovarian puncture method (Figure 4A). After intracytoplasmic sperm injection (ICSI), seven zygotes developed into transferable embryos, and five blastocysts were biopsied for amplification with multiple annealing and looping-based cycles (Figure 4B).

The linkage analysis with SNP array for haplotype showed that two embryos were normal, and the other three were affected (Figure 4C). Finally, one healthy embryo was transferred, and luteal phase supports were administered routinely. Serum β-hCG levels were measured at 14 days after frozen embryo transplantation (FET). The presence of a gestational sac and fetal heartbeat detected by ultrasound at 5 weeks after FET was evidence of clinical pregnancy. Sanger sequencing was performed on the amniotic fluid sample collected by amniocentesis at 18 weeks of gestation; no *MKS1* mutation was found.

## Discussion

In this study, we reported a Chinese MKS-related family with two novel *MKS1* mutations displaying occipital encephalocele, cerebellar vermis agenesis, and postaxial polydactyly. No renal/hepatic involvement was observed by ultrasonography. The nonsense mutation, c.350C>A, induced premature termination of MKS1 translation but did not trigger the degradation of mutant mRNA by NMD. The c.1408-14A>G variant was in intron 15 and resulted in the skip of exon 16 during the *MKS1* mRNA splice, thereby coding an elongated MKS1 protein (p.E471Lfs*92). *In vitro* functional analysis with 293T cells showed the instability of MKS1 p.S117* and the disruption of B9-C2 domain in MKS1 p.E471Lfs*92. Finally, the non-consanguineous couple was assisted with PGT-M for pregnancy with a healthy baby without *MKS1* mutations.

The *MKS1*-related genotype–phenotype correlation was proposed as follows: two null alleles of *MKS1* result in MKS; one null allele and one non-truncating allele that leaves the B9-C2 domain intact result in JBTS; two non-truncating alleles result in Bardet–Biedl syndrome (BBS, MIM 615990) (Bader et al., 2016; Luo et al., 2020). Previous studies have identified the compound heterozygous mutations of MKS1 (p.R158* and p.E471Lfs*92), which disrupted the intracellular localization of MKS1 and induced defects in cilium length and the number of patient fibroblasts (Slaats et al., 2016). In the present study, the two variants (p.S117* and p.E471Lfs*92) identified in the MKS-related fetuses localized near the reported mutations and functioned through the comparable genotype–phenotype regulation model.

According to Mendel’s law of inheritance, the incidence of autosomal recessive diseases in the offspring is 25%. Moreover, the frequency of genetic mutations is variable among the populations in different regions. In the assisted reproductive process, high attention is focused on preventing genetic diseases, especially autosomal recessive diseases. As a well-established alternative to invasive prenatal diagnosis, PGT for monogenic disorder (PGT-M) has evolved into an effective clinical method for MKS-related families.

In summary, we identified two novel variants, expanding the mutation spectrum of *MKS1*. Our findings further implicated that the clinical significance of *MKS1* variants needs an in-depth investigation. PGT and extended carrier screening are effective tools for genetic disease blocking in clinical applications. Together, these findings would be beneficial for the MKS patients and their families.

## Data Availability

The data presented in the study are deposited in the Genome Sequence Archive for Human in China National Genomics Data Center repository, accession number HRA001710, that are publicly accessible at https://ngdc.cncb.ac.cn/gsa-human.
