# Supplementary material for: Case Report: Preimplantation Genetic Testing for Meckel Syndrome Induced by Novel Compound Heterozygous Mutations of MKS1
Source: Front Genet. 2022 Mar 14;13:843931. doi: 10.3389/fgene.2022.843931 (PMC8963843; doi:10.3389/fgene.2022.843931)
Supplement: Supplementary file 1 [file Table1.DOCX]

| **Table S1. Clinical features and genotype of the MKS-related fetuses.** | | | |
| --- | --- | --- | --- |
| Patient ID | Ⅱ:1 | Ⅱ:2 | Ⅱ:3 |
| Chromosome karyotype | 46, XX | 46, XY | ND |
| Year | 2017 | 2018 | 2020 |
| Diagnosed time | 13w2d | 12w5d | 16w5d |
| Ethnicity | Chinese | Chinese | Chinese |
| Mutation1 | c.350C>A | c.350C>A | c.350C>A |
| Mutation2 | c.1408-14A>G | c.1408-14A>G | c.1408-14A>G |
| Occipital encephalocele | + | + | + |
| enlarged fourth ventricle | + | + | + |
| agenesis of the corpus callosum | + | + | + |
| postaxial polydactyly | + | + | + |
| cystic renal dysplasia^*^ | - | - | - |
| cleft lip and palate | - | - | - |
| fibrotic changes of the liver | - | - | - |

*: The clinical features were observed by ultrasonography.
