# Supplementary material for: Case Report: Preimplantation Genetic Testing for Meckel Syndrome Induced by Novel Compound Heterozygous Mutations of MKS1
Source: Front Genet. 2022 Mar 14;13:843931. doi: 10.3389/fgene.2022.843931 (PMC8963843; doi:10.3389/fgene.2022.843931)
Supplement: Supplementary file 2 [file Table2.DOCX]

**Table S2. The 22 variants have been identified at the 3’-terminus of intron 15.**

| **Num** | **Chr** | **Position** | **rsIDs** | **Ref** | **Alt** | **Transcript Consequence** | **VEP**  **Annotation** | **ClinVar Clinical Significance** | **varSEAK Online Effect** | **SpliceAI Score (Acceptor Loss)** | **Allele Count** | **Allele Number** | **Allele**  **Frequency** |
| --- | --- | --- | --- | --- | --- | --- | --- | --- | --- | --- | --- | --- | --- |
| 1 | 17 | 56283866 | rs1473936553 | C | T | c.1408-1G>A | splice_acceptor_variant |  | Exon skipping | 0.99 | 1 | 249146 | 4.01371E-06 |
| 2 | 17 | 56283911 | rs377033273 | G | A | c.1408-3C>T | splice_region_variant | Uncertain significance | Exon skipping | 0.01 | 19 | 277988 | 6.83483E-05 |
| 3 | 17 | 56283912 | rs552287204 | T | G | c.1408-4A>C | splice_region_variant |  | No splicing effect. | 0.01 | 1 | 246436 | 4.05785E-06 |
| 4 | 17 | 56283912 | rs552287204 | T | C | c.1408-4A>G | splice_region_variant |  | Exon skipping | 0.03 | 2 | 246436 | 8.1157E-06 |
| 5 | 17 | 56283913 | rs1237002420 | GGCATGCCA  TTGGGACAG  CCTCAGGTTTCT | G | c.1408-34_1408-6del | splice_region_variant | Conflicting interpretations of pathogenicity | Exon skipping | 0.99 | 308 | 277812 | 0.00110866341266755 |
| 6 | 17 | 56283922 | rs1194131222 | T | C | c.1408-14A>G | intron_variant |  | Likely no splicing effect. | 0.40 | 2 | 276710 | 7.22778E-06 |
| 7 | 17 | 56283923 | rs762989365 | T | C | c.1408-15A>G | intron_variant | Conflicting interpretations of pathogenicity | Likely no splicing effect. | 0.00 | 6 | 245096 | 2.44802E-05 |
| 8 | 17 | 56283928 | rs1167242242 | C | T | c.1408-20G>A | intron_variant |  | Likely no splicing effect. | 0.00 | 1 | 244322 | 4.09296E-06 |
| 9 | 17 | 56283929 |  | A | C | c.1408-21T>G | intron_variant |  | Likely no splicing effect. | 0.00 | 1 | 244190 | 4.09517E-06 |
| 10 | 17 | 56283931 | rs766480492 | C | A | c.1408-23G>T | intron_variant |  | No splicing effect. | 0.01 | 1 | 243588 | 4.10529E-06 |
| 11 | 17 | 56283934 | rs752001284 | C | G | c.1408-26G>C | intron_variant |  | No splicing effect. | 0.01 | 1 | 242900 | 4.11692E-06 |
| 12 | 17 | 56283936 | rs1374491789 | G | C | c.1408-28C>G | intron_variant |  | Likely no splicing effect. | 0.02 | 5 | 242698 | 2.06017E-05 |
| 13 | 17 | 56283946 | rs369698284 | C | T | c.1408-38G>A | intron_variant |  | No splicing effect. | 0.00 | 1 | 239928 | 4.16792E-06 |
| 14 | 17 | 56283946 | rs369698284 | C | G | c.1408-38G>C | intron_variant |  | No splicing effect. | 0.00 | 41 | 271304 | 0.000151122 |
| 15 | 17 | 56283957 | rs200222851 | C | T | c.1408-49G>A | intron_variant |  | No splicing effect. | 0.01 | 2 | 235492 | 8.49286E-06 |
| 16 | 17 | 56283957 | rs200222851 | C | G | c.1408-49G>C | intron_variant | Likely benign | No splicing effect. | 0.00 | 289 | 266858 | 0.001082973 |
| 17 | 17 | 56283958 | rs778481228 | C | G | c.1408-50G>C | intron_variant |  | No splicing effect. | 0.21 | 1 | 235318 | 4.24957E-06 |
| 18 | 17 | 56283958 | rs778481228 | C | A | c.1408-50G>T | intron_variant |  | No splicing effect. | 0.13 | 2 | 235318 | 8.49914E-06 |
| 19 | 17 | 56283960 | rs745555659 | C | T | c.1408-52G>A | intron_variant |  | No splicing effect. | 0.16 | 1 | 234586 | 4.26283E-06 |
| 20 | 17 | 56283961 | rs539223121 | C | T | c.1408-53G>A | intron_variant |  | No splicing effect. | 0.13 | 5 | 31374 | 0.000159368 |
| 21 | 17 | 56283962 | rs557644685 | G | A | c.1408-54C>T | intron_variant |  | No splicing effect. | 0.24 | 3 | 31366 | 9.5645E-05 |
| 22 | 17 | 56283980 | rs1348802765 | C | A | c.1408-72G>T | intron_variant |  | No splicing effect. | 0.07 | 1 | 31384 | 3.18634E-05 |
