# Supplementary material for: Case Report: Preimplantation Genetic Testing for Meckel Syndrome Induced by Novel Compound Heterozygous Mutations of MKS1
Source: Front Genet. 2022 Mar 14;13:843931. doi: 10.3389/fgene.2022.843931 (PMC8963843; doi:10.3389/fgene.2022.843931)
Supplement: Supplementary file 3 [file DataSheet1.PDF]

Check out the Mutalyzer 3 Alpha (https://v3.mutalyzer.nl/) release!

Name Checker

Please insert a variant description using the HGVS (http://varnomen.hgvs.org/) format.

Variant description

NM_017777.4(MKS1_v001):c.1408_1490del

Examples: AB026906.1:c.40_42del , NG_012337.1(SDHD_v001):c.274G>T , LRG_24t1:c.159dup

Check variant description

Help (https://github.com/mutalyzer/mutalyzer/wiki/Name-Checker)

Removed 2 splice sites from selected transcript.

Sequence "GGGGAA [71bp] GTCCAG" at position 1433_1515 was not corrected to "GGAACG [71bp] CCAGGG" at position 1435_1517, since they reside in different exons.

0 Errors, 0 Warnings.

Overview of the raw variants

Raw variant 1: deletion of 1433 to 1515

```
TGTACGGATACCAGGATCCTTCAAG GGGGAA [71bp] GTCCAG GGCCTTCATGGAATCGAGCTCCCTT
TGTACGGATACCAGGATCCTTCAAG -------------------- GGCCTTCATGGAATCGAGCTCCCTT
```

Description relative to transcription start

(Not for use in LSDBs in case of protein-coding transcripts).

NM_017777.4:n.1433_1515del (/name-checker?description=NM_017777.4%3An.1433_1515del)

Affected transcripts - 1

NM_017777.4(MKS1_v001):c.1408_1490del (/name-checker?description=NM_017777.4%28MKS1_v001%29%3Ac.1408_1490del)

Affected proteins - 1

```
NM_017777.4(MKS1_i001):p.(Glu471Leufs*92)
```

Reference protein

```
  1 MAETVWSTDT GEAVYRSRDP VRNLRLRVHL QRITSSNFLH YQPAAELGKD LIDLATFRPQ
 61 PTASGHRPEE DEEEEIVIGW QEKLFSQFEV DLYQNETACQ SPLDYQYRQE ILKLENSGGK
121 KNRRIFTYTD SDRYTNLEEH CQRMTTAASE VPSFLVERMA NVRRRRQDRR GMEGGILKSR
181 IVTWEPSEEF VRNNHVINTP LQTMHIMADL GPYKKLGYKK YEHVLCTLKV DSNGVITVKP
241 DFTGLKGPYR IETEGEKQEL WKYTIDNVSP HAQPEEEERE RRVFKDLYGR HKEYLSSLVG
301 TDFEMTVPGA LRLFVNGEVV SAQGYEYDNL YVHFFVELPT AHWSSPAFQQ LSGVTQTCTT
361 KSLAMDKVAH FSYPFTFEAF FLHEDESSDA LPEWPVLYCE VLSLDFWQRY RVEGYGAVVL
421 PATPGSHTLT VSTWRPVELG TVAELRRFFI GGSLELEDLS YVRIPGSFKG ERLSRFGLRT
481 ETTGTVTFRL HCLQQSRAFM ESSSLQKRMR SVLDRLEGFS QQSSIHNVLE AFRRARRRMQ
541 EARESLPQDL VSPSGTLVS*
```

Protein predicted from variant coding sequence

```
  1 MAETVWSTDT GEAVYRSRDP VRNLRLRVHL QRITSSNFLH YQPAAELGKD LIDLATFRPQ
 61 PTASGHRPEE DEEEEIVIGW QEKLFSQFEV DLYQNETACQ SPLDYQYRQE ILKLENSGGK
121 KNRRIFTYTD SDRYTNLEEH CQRMTTAASE VPSFLVERMA NVRRRRQDRR GMEGGILKSR
181 IVTWEPSEEF VRNNHVINTP LQTMHIMADL GPYKKLGYKK YEHVLCTLKV DSNGVITVKP
241 DFTGLKGPYR IETEGEKQEL WKYTIDNVSP HAQPEEEERE RRVFKDLYGR HKEYLSSLVG
301 TDFEMTVPGA LRLFVNGEVV SAQGYEYDNL YVHFFVELPT AHWSSPAFQQ LSGVTQTCTT
361 KSLAMDKVAH FSYPFTFEAF FLHEDESSDA LPEWPVLYCE VLSLDFWQRY RVEGYGAVVL
421 PATPGSHTLT VSTWRPVELG TVAELRRFFI GGSLELEDLS YVRIPGSFKG LHGIELPSEK
481 DAECVGPSGR VQPAEFHSQC ARGLPSSPAP HAGGPGKPPA GPSEPLWNPG LLAHSSPGPQ
541 CKRTRWGISE ASALLPLRLS D*
```

Effects on Restriction sites

Raw variant	Created	Deleted
1		AciI (3), AlwNI, ApeKI, BbvI, BcoDI, Bpu10I, BsaI, BsaJI, BslI, BsmAI, BspCNI, BstAPI, BtsIMutI (2), Cac8I, DdeI, Fnu4HI (2), HphI, HpyCH4III (2), HpyCH4V (2), MlyI, MwoI (2), PleI, PstI, SfcI, TseI, Tsp45I, TspRI (2), XcmI

Experimental services

Genomic description: `1435_1517del`

Exon information

Number	Start (g.)	Stop (g.)	Start (c.)	Stop (c.)
1	1	105	-25	80
2	106	215	81	190
3	216	286	191	261
4	287	442	262	417

Number	Start (g.)	Stop (g.)	Start (c.)	Stop (c.)
5	443	540	418	515
6	541	669	516	644
7	670	774	645	749
8	775	883	750	858
9	884	940	859	915
10	941	983	916	958
11	984	1049	959	1024
12	1050	1120	1025	1095
13	1121	1190	1096	1165
14	1191	1298	1166	1273
15	1299	1432	1274	1407
16	1433	1515	1408	1490
17	1516	1613	1491	1588
18	1614	2343	1589	*638

CDS information

	g.	c.
Start	26	1
Stop	1705	1680

Links

Download this reference sequence file: NM_017777.4.gb (/reference/NM_017777.4.gb)

Legend

© 2009-2021 LUMC (http://www.lumc.nl)
Disclaimer (https://www.gnu.org/licenses/agpl-3.0.html)
